# Trans-pacific multicenter collaborative study of minimally invasive proximal versus total gastrectomy for proximal gastric and gastroesophageal junction cancers

**DOI:** 10.1186/s12893-023-02163-8

**Published:** 2023-09-01

**Authors:** Naruhiko Ikoma, Travis Grotz, Hirofumi Kawakubo, Hyoung-Il Kim, Satoru Matsuda, Yuki Hirata, Atsushi Nakao, Loretta A. Williams, Xin Shelley Wang, Tito Mendoza, Xuemei Wang, Brian D. Badgwell, Paul F. Mansfield, Woo-Jin Hyung, Vivian E. Strong, Yuko Kitagawa

**Affiliations:** 1 Department of Surgical Oncology, The University of Texas MD Anderson Cancer Center, Houston, TX USA; 2 Mayo Clinic, Rochester, MN USA; 3 Keio University School of Medicine, Tokyo, Japan; 4 Department of Surgery, Yonsei University College of Medicine, 50-1 Yonsei-Ro, Seodaemun-Gu, Seoul, 03722 Republic of Korea; 5 Department of Symptom Research, The University of Texas MD Anderson Cancer Center, Houston, TX USA; 6 Department of Biostatistics, The University of Texas MD Anderson Cancer Center, Houston, TX USA; 7 Department of Surgery, Memorial Sloan Kettering Cancer Center, New York, NY USA

**Keywords:** Robotic gastrectomy, Proximal gastrectomy, Total gastrectomy, Patient-reported outcomes, Quality of life

## Abstract

**Background:**

The current standard operation for proximal gastric and gastroesophageal junction (P/GEJ) cancers with limited esophageal extension is total gastrectomy (TG). TG is associated with impaired appetite and weight loss due to the loss of gastric functions such as production of ghrelin and with anemia due to intrinsic factor loss and vitamin B_12_ malabsorption. Theoretically, proximal gastrectomy (PG) can mitigate these problems by preserving gastric function. However, PG with direct esophagogastric reconstruction is associated with severe postoperative reflux, delayed gastric emptying, and poor quality of life (QoL). Minimally invasive PG (MIPG) with antireflux techniques has been increasingly performed by experts but is technically demanding owing to its complexity. Moreover, the actual advantages of MIPG over minimally invasive TG (MITG) with regards to postoperative QoL are unknown. Our overall objective of this study is to determine the short-term QoL benefits of MIPG. Our central hypotheses are that MIPG is safe and that patients have improved appetite after MIPG with effective antireflux techniques, which leads to an overall QoL improvement when compared with MITG.

**Methods:**

Enrollment of a total of 60 patients in this prospective survey-collection study is expected. Procedures (MITG versus MIPG, antireflux techniques for MIPG [double-tract reconstruction versus the double-flap technique]) will be chosen based on surgeon and/or patient preference. Randomization is not considered feasible because patients often have strong preferences regarding MITG and MIPG. The primary outcome is appetite level (reported on a 0-10 scale) at 3 months after surgery. With an expected 30 patients per cohort (MITG versus MIPG), this study will have 80% power to detect a one-point difference in appetite level. Patient-reported outcomes will be longitudinally collected (including questions about appetite and reflux), and specific QoL items, body weight, body mass index and ghrelin, albumin, and hemoglobin levels will be compared.

**Discussion:**

Surgeons from the US, Japan, and South Korea formed this collaboration with the agreement that the surgical approach to P/GEJ cancers is an internationally important but controversial topic that requires immediate action. At the completion of the proposed research, our expected outcome is the establishment of the benefit and safety of MIPG.

**Trial registration:**

This trial was registered with Clinical Trials Reporting Program Registration under the registration number NCI-2022–00267 on January 11, 2022, as well as with ClinicalTrials.gov under the registration number NCT05205343 on January 11, 2022.

## Background

The current standard surgical procedure used to treat proximal gastric and gastroesophageal junction (P/GEJ) cancers with limited esophageal extension is total gastrectomy (TG), unless the tumor is small (< 3 cm in diameter) and contained in the mucosal layer (pT1a); such tumors can be removed via endoscopic mucosal resection [1]. TG is associated with impaired appetite and weight loss [2–4] due to the loss of gastric functions such as production of ghrelin (the “hunger hormone” secreted by the stomach) [5] and with anemia due to intrinsic factor loss and vitamin B_12_ malabsorption. Theoretically, proximal gastrectomy (PG) can mitigate these problems by preserving gastric function. However, PG with direct esophagogastric reconstruction is associated with severe postoperative reflux and poor quality of life (QoL) and is therefore rarely performed in Western countries, including the United States [6, 7]. The lack of an effective surgical procedure that preserves gastric function and postoperative QoL for patients with P/GEJ cancers is a critical gap.

With the recent development of effective post-PG anti-reflux reconstruction techniques, such as double-tract reconstruction and the double-flap technique, PG has been increasingly used to treat proximal gastric cancer in South Korea and Japan [8–13]. Researchers in the KLASS-05 trial (NCT02892643) have recently reported results comparing outcomes at 2 years after PG with outcomes at 2 years after TG in patients with cT1N0 proximal gastric cancer, and these results showed that PG reduced the need for vitamin B_12_ supplementation and improved some QoL measurements. However, it remains unknown whether PG with double-tract reconstruction or the double-flap technique has short-term postoperative QoL advantages over TG [8, 12, 13]. In addition, the theoretical disadvantages of PG include incomplete lymph node (LN) removal, which may result in recurrence, and the potential for a second gastric cancer in the remnant stomach. Moreover, delayed gastric emptying of the remnant stomach can cause upper gastrointestinal symptoms such as reflux and bloating. The QoL benefits of PG must therefore be clearly demonstrated before encouraging its use in Western countries.

PG is commonly indicated for cT1 proximal gastric cancer in South Korea and Japan [8–13], with reported excellent overall and recurrence-free survival. Given the low incidence of LN metastasis in cT1 gastric cancers, PG is considered safe for cT1 proximal gastric cancer. A recent Japanese prospective study of GEJ adenocarcinoma reported a low incidence of LN metastasis at peripyloric stations (suprapyloric [station 5] and infrapyloric [station 6]) regardless of tumor stage [14], and surgeons thus began performing PG for GEJ cancers in Eastern Asia. As a result, Japanese Gastric Cancer Treatment Guidelines now recommend PG as a surgical option for GEJ cancers with limited esophageal extension [15]. In Western countries, although TG is considered the standard surgical procedure for P/GEJ cancers, Ivor Lewis esophagectomy, which uses the gastric conduit for reconstruction [16], is also commonly performed for GEJ cancers, suggesting that PG may also be oncologically safe for GEJ cancers. However, internationally and nationally, controversy remains as to whether and when PG is oncologically safe for GEJ cancers and whether PG provides better QoL than TG [6, 7]. An international collaboration is warranted to determine the benefits and safety of PG for P/GEJ cancers and thus standardize surgical care for patients with these cancers.

### Rationale

Surgeons at The University of Texas MD Anderson Cancer Center in Houston, Texas; the Mayo Clinic in Rochester, Minnesota; Memorial Sloan Kettering Cancer Center, New York; Yonsei University in Seoul, South Korea; and Keio University in Tokyo, Japan formed a collaboration to address the issue of the surgical approach for P/GEJ cancers, an internationally important but controversial topic that requires immediate action. Our central hypotheses are that PG is safe and that patients have improved levels of appetite after minimally invasive PG (MIPG) with effective anti-reflux techniques because of maintained secretion of ghrelin from the gastric remnant, which leads to better overall QoL compared with minimally invasive TG (MITG). The rationale for this project is that MIPG’s theoretical benefit of preserving gastric function, which can only be measured using patient-reported outcomes (PROs), must be demonstrated before widespread implementation of MIPG.

### Study objectives

The primary objective is to compare the short-term appetite of patients who undergo MIPG for gastric and gastroesophageal adenocarcinoma with that of patients who undergo MITG. We hypothesize that MIPG is associated with higher postoperative appetite levels compared with MITG, which would result in better nutritional status and less body weight loss after surgery.

The secondary objective is to assess PROs and nutrition measures and compare them between the MIPG and MITG groups. We will use the MD Anderson Symptom Inventory Gastrointestinal Cancer Module (MDASI-GI) questionnaire, along with an additional three experimental question items (MDASI-GI +), to collect preoperative and postoperative PRO measures of QoL, and we will check fasting ghrelin levels to correlate them with reported appetite levels. We will also investigate factors associated with improved QoL after surgery, safety of MIPG and MITG, and oncologic outcomes after MIPG and MITG.

## Methods/design

This is a multicenter, open-label, prospective cohort study comparing outcomes of MIPG (trial procedure) and MITG (standard of care, control group). We expect to enroll a total of 60 patients in this study (30 per group). Patients will be recruited from and treated at MD Anderson Cancer Center, Mayo Clinic, Memorial Sloan Kettering Cancer Center, Yonsei University, and Keio University, after Institutional Review Board approval for each institution. We plan to enroll at least 20 patients each from Asian (Keio University and Yonsei University) and US (MD Anderson, and Mayo Clinic Minnesota, and Memorial Sloan Kettering Cancer Center) institutions. The total study duration is expected to be 60 months (including enrollment and follow-up). We will prospectively collect PROs longitudinally for a 1-year period. Other clinical factors will also be collected to investigate factors associated with improved PROs.

### Participants

#### Inclusion criteria

1. Able to speak and read English, Spanish, Korean, or Japanese
2. Biopsy-confirmed diagnosis of non-metastatic gastric or GEJ adenocarcinoma scheduled to undergo curative-intent MIPG or MITG
3. Age ≥ 18

#### Exclusion criteria

1. Known malabsorption syndrome or a lack of physical integrity of the upper gastrointestinal tract
2. Known narcotic dependence, with average daily dose > 5 mg oral morphine equivalent
3. Unable to comply with study and/or follow-up procedures, deemed as such at investigators’ discretion
4. Pregnant (thus excluded from receiving MIPG or MITG)

#### Recruitment

After being screened for inclusion and exclusion criteria, the potential participants will be verbally introduced to the study by investigators. Each participant will sign a written informed consent form before enrollment into the study. A copy of the signed consent form will be given to the participant, and the original consent form will be kept in the system at each hospital.

### Interventions

#### Preoperative evaluation

**Fig. 1:**
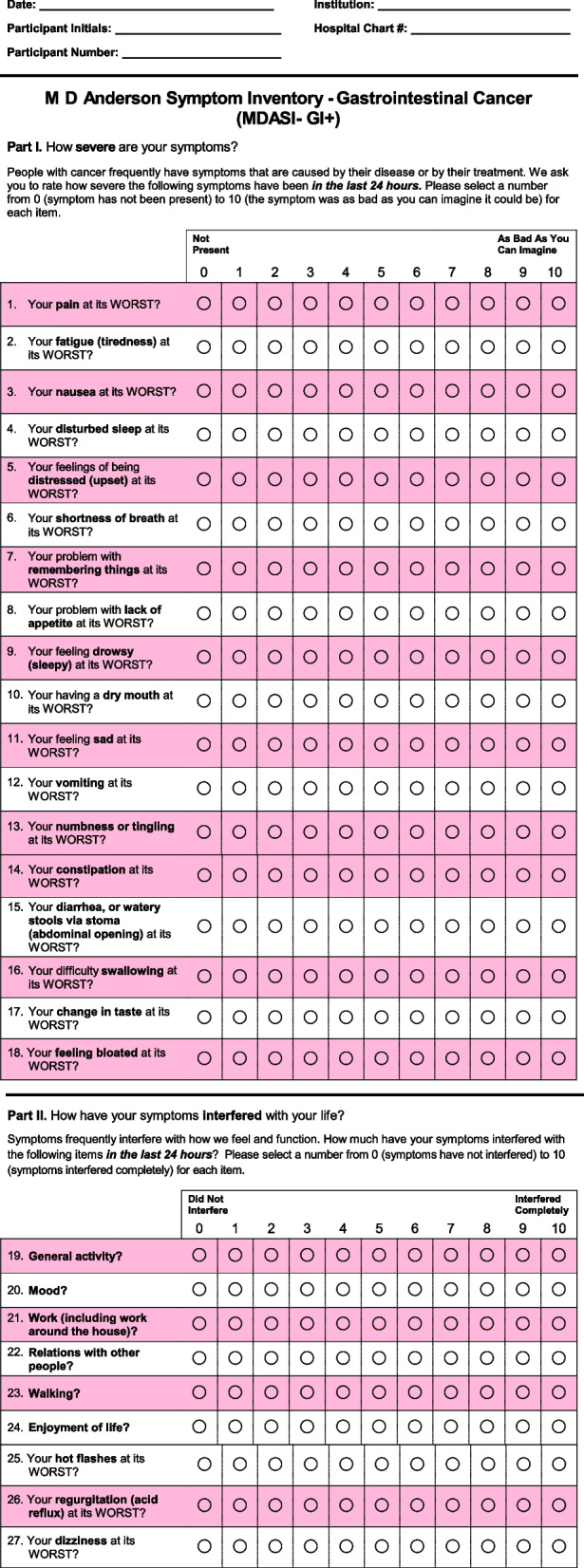
M. D. Anderson Symptom Inventory – Gastrointestinal Cancer (MDASI-GI +)

Within 30 days prior to surgery, PRO collection will be completed using the MDASI-GI + (Fig. 1). This can be done by paper or electronic-based communication (REDCap).

#### Details of surgery

**Fig. 2:**
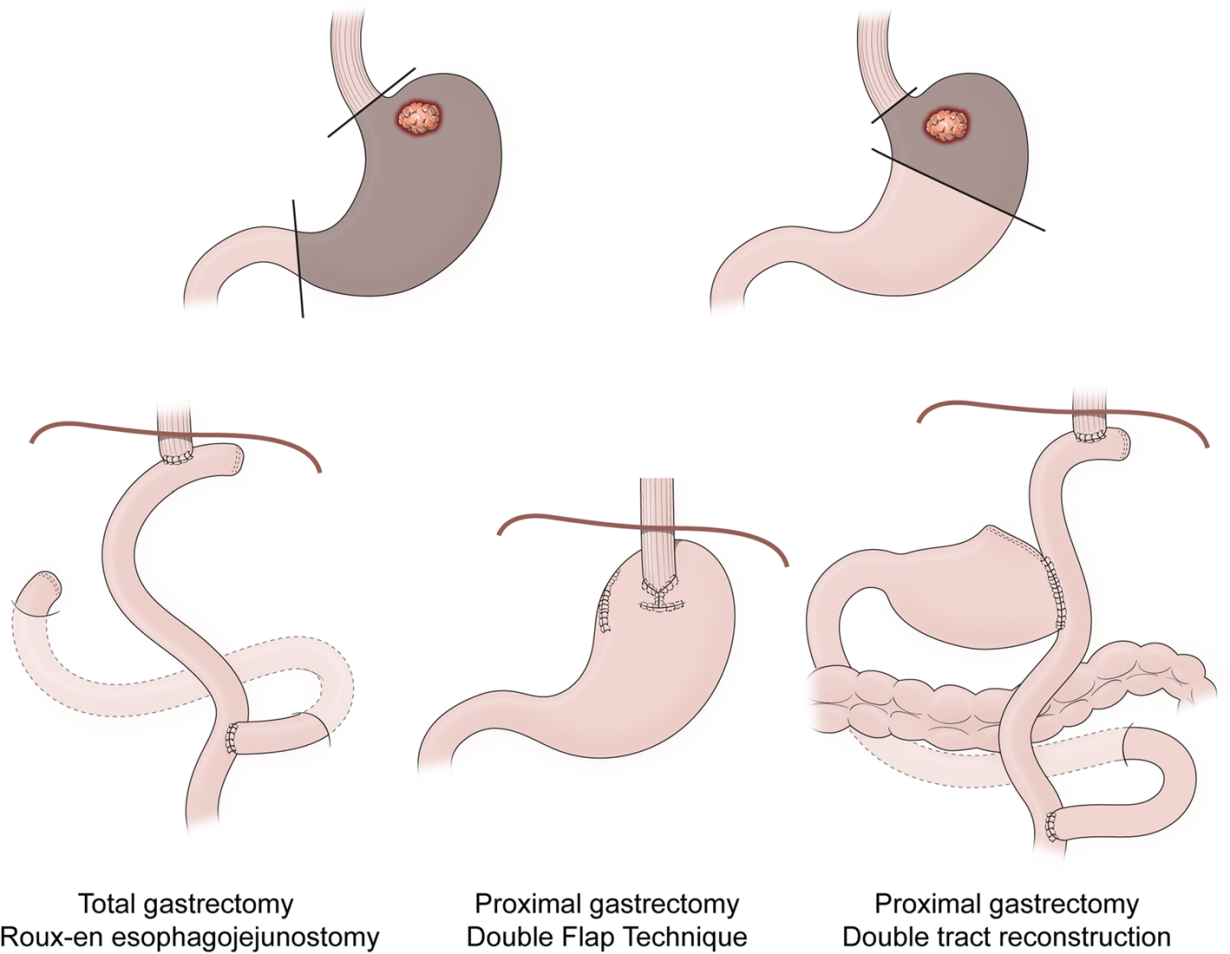
Techniques of reconstructions after total and proximal gastrectomy

After adequate preoperative evaluation, patients who are judged to be physiologically capable of undergoing resection, viewed as having resectable lesions, and without evidence of metastatic disease will undergo MITG or MIPG as per their choice and the surgeon’s discretion. We do not consider randomization feasible because patients often have strong preferences regarding MITG and MIPG. After MITG, a Roux-en-Y reconstruction is performed, whereas after MIPG, an anti-reflux reconstruction technique is chosen by the surgeon (double-tract reconstruction [17] or the double-flap technique [18]) (Fig. 2). All participating surgeons need to have performed at least 30 robotic gastrectomies before enrolling patients into the study, and at least 10 of these procedures need to have used the specific type of reconstruction technique (e.g., double-flap reconstruction after proximal gastrectomy) that the patient will receive. Details of the reconstruction techniques are determined by the surgeon. Patients will undergo standard postgastrectomy care at each institution during their hospital stays. A feeding jejunostomy can be placed for postoperative nutritional support at the surgeon’s discretion. These surgical procedures can be performed with a laparoscopic or robotic approach. Patients who undergo conversion to open laparotomy will be excluded from the study analyses, since conversion is expected to impact postoperative recovery. Patients who are found to have metastatic disease during surgery will also be excluded from the study analyses, regardless of whether the patient completed gastrectomy or not. Those excluded from the study analyses will be considered screening failures to allow for additional enrollees to meet the accrual target.

#### Perioperative management, discharge, and follow-up

After being discharged from the hospital, patients will be followed up at the surgical clinic for PRO collection, which can be done in person or virtually. Other clinical outcomes such as nutritional assessment, disease recurrence, and survival will be collected retrospectively through medical records.

#### Posttreatment outpatient evaluation

Post-discharge follow-up evaluations for PRO collection will occur at 1 month (± 2 weeks) and 3, 6, and 12 months (± 1 month) after surgery. These may be performed in person or by telephone, email, or website-based reporting system (REDCap), and data will be subsequently stored in the REDCap database, which will be managed at MD Anderson (center institution of this study).

### Outcome measures

The primary endpoint is appetite level (reported on a 0–10 scale, in Q8 of MDASI-GI +) at 3 months after surgery. The individual and total MDASI-GI + scores will also be reported at baseline (preoperative) and 1, 3, 6, and 12 months after surgery.

The secondary endpoints below will be collected retrospectively from the medical record.

#### Nutritional measures

Body weight and serum levels of albumin, ferritin, vitamin B_12_, hemoglobin, and ghrelin will be measured at baseline and 1, 3, 6, and 12 months after surgery.

#### Safety measures

The rate and types of postoperative complications within 90 days after surgery, length of post-operative hospital stay, and rate of post-discharge re-admission within 90 days after surgery will be recorded.

#### Oncologic measures

The incidence of loco-regional recurrence and death within 3 years after MITG or MIPG will be recorded.

#### Additional clinical data

The following data will be collected retrospectively if available in the medical record: treating institution (MD Anderson, Mayo Clinic, Memorial Sloan Kettering, Yonsei, or Keio), patient age, gender, race/ethnicity, body weight, height, use of concurrent medications such as opioids, initial diagnosis date, date of surgery, tumor size and location (diameter and esophageal extension above GEJ, Siewert classification, by endoscopic assessment), preoperative imaging (computed tomography, positron emission tomography, magnetic resonance imaging, endoscopic ultrasound) findings, type of preoperative therapy, laparoscopic findings, operating room time and blood loss, postoperative morbidity and mortality within 90 days after surgery, length of hospital stay, incidence of re-admission within 90 days after surgery, pathologic details (histologic type, TNM category, margin status), serum laboratory data (hemoglobin, leukocytes, absolute neutrophil count, platelet count, blood urea nitrogen, creatinine, albumin, pre-albumin, ghrelin, ferritin, vitamin B_12_) and date collected, recurrent disease (incidence, date, and location), and date of last follow-up and vital status.

The MDASI-GI is a validated questionnaire for measuring symptom severity and interference with function in patients with gastrointestinal cancers, including gastric cancer [19]. Using this questionnaire, patients score the severity of their symptoms over the previous 24 h on a scale of 0 (not present) to 10 (as bad as you can imagine). To further investigate patient symptom burden after gastrectomy, three additional questions, about the presence and degree of reflux, hot flashes, and dizziness, were added to the MDASI-GI to create the MDASI-GI + (Fig. 1). The rationale for adding these three questions is that acid and bilious reflux and dumping syndrome are two of the most common symptoms patients experience after major surgery for upper gastrointestinal cancers. Hot flashes and dizziness are frequently experienced with dumping syndrome.

### Translation of the MDASI-GI + into Korean, Japanese, and Spanish

The majority of the MDASI-GI questionnaire items were available in Spanish, Korean, and Japanese, but the three additional questions [items 25–27] had not been translated into the three languages and items 15–18 had not been translated into Japanese. We followed a standardized translation process as described below.

1. Two translators who are fluent in both the requested language and English and who worked separately translated the text.
2. One translator translated the written English items into the requested language in writing. This translation followed the established format, wording, and style of the rest of the MDASI translated into that language. The first translator was given a copy of the core MDASI in the requested language to facilitate this process.
3. The second translator translated the written requested language items back into English in writing.
4. The PI summarized the translation process and asked an experienced collaborator in the Symptom Management Department at MD Anderson to review and approve the written translations from English to the requested language and back to English. If the translations did not adequately convey the intended meaning of the items, we asked for new translations into the requested language and then back translations into English until a satisfactory translation was achieved.
5. Approved translations were tested in 5 patients fluent in the translated language to confirm that the translated questions are easy to understand without creating confusion.

### Statistical methods

The primary outcome is appetite level score (with 0 being the best and 10 being the worst) according to the MDASI-GI + (Question #8) at 3 months after surgery. We will enroll a total of 64 patients in order to have 60 evaluable patients at 3 months after surgery (i.e., assuming a 5% attrition rate). With 30 patients expected in each cohort, this study will have 80% power to detect a one-point difference in appetite level score using a two-sample *t*-test with a 5% significance level and assuming a common standard deviation of 1.35 (nQuery Advisor 7.0). We will also compare serum ghrelin levels at 3 months after surgery. For the primary analyses, we will apply the inverse probability treatment weights using propensity scores for assessing the difference between the two surgical groups. Inverse probability weighting is applied by weighting the outcome measures by the inverse of the probability of an individual with a given set of covariates being assigned to their treatment (i.e., propensity score). The propensity scores will be derived through fitting a multiple logistic regression model with MIPG (vs. MITG) as the outcome variable. Then, in the presence of measured confounders, we can estimate the difference between the two treatment groups via inverse probability weighting of the study subjects. For the regression analyses described below for assessing the “surgical procedure” effect, the inverse probability treatment weights using propensity scores will be applied as appropriate in addition to any standard statistical methods. The two-sample t-test or Wilcoxon rank-sum test will also be used to compare the appetite level or ghrelin level at 3 months, as well as the percent change from baseline, between the two cohorts. We will longitudinally collect PROs (using the fully translated MDASI-GI +) and compare specific items and overall scores as well as body weight, body mass index, and albumin and hemoglobin levels at 1, 3, 6, and 12 months after surgery in the cohorts. A linear mixed effects model will be fit to assess the change in QoL over time while accounting for potential intra-patient correlations. “Time” points will be included as a fixed effect, and “patient” will be included as a random effect. The surgical procedure received and use of adjuvant or neoadjuvant therapy will be included as covariates. Similar analyses will be performed to assess the changes in body weight, body mass index, and albumin and hemoglobin levels over time. The number of LNs examined pathologically, incidence of postoperative complications, operating room time, blood loss, and length of hospital stay will be compared. Continuous variables (such as length of hospital stay) will be assessed using the two-sample t-test or Wilcoxon rank-sum test. Categorical variables (such as type and severity of complications) will be assessed using Fisher’s exact test. Local–regional recurrence-free and overall survival will be estimated using the Kaplan–Meier method, and Cox regression analyses will be performed to assess the association with patient characteristics and surgical procedure received. The 3-year loco-regional recurrence rate will be estimated along with the 95% confidence interval. Missing data will be excluded from analysis and no missing data imputation will be performed. Sensitivity analyses to investigate factors associated with PRO scores, including but not limited to country (west vs. east), tumor location, types of reconstruction after PG, use of neoadjuvant and adjuvant therapy, use of jejunostomy or nutrition status, on PRO scores will be performed.

### Dissemination policy

This trial is intended for publication in international peer-reviewed journals. The results of this study will also be presented at internationally relevant scientific meetings. The progress and the results of the study will be saved at Clinicaltrials.gov to allow general access to documented findings.

## Discussion

Despite the increasing incidence of proximal gastric cancer and P/GEJ cancers [20], the optimal treatment strategy for those cancers, including regimens of adjuvant and neoadjuvant therapy and extent of gastrectomy (such as when a transthoracic approach or TG is needed), remains unknown.

The optimal operation for P/GEJ cancers should achieve complete removal of the primary cancer and regional LNs that harbor risks of metastases while maintaining the best possible QoL for the patient. It should also facilitate a quick return to intended oncologic therapy such as adjuvant chemotherapy. In this context, it is unknown whether and the extent to which PG would result in better QoL than TG.

Kunisaki et al. conducted a PGSAS NEXT survey study of gastric cancer patients who underwent TG or PG [21]. This study utilized a questionnaire developed for postgastrectomy patients in Japan (PGSAS-45), which was validated in patients with stage I gastric cancer without receipt of adjuvant or neoadjuvant therapy [22]. PGSAS-45 consists of 45 question items, and 19 subscale “main outcome measures” are calculated on the basis of responses. The PGSAS NEXT study enrolled 1,020 TG patients and 518 PG patients. The PG patients had better scores in several main outcome measures (weight loss, dumping syndrome, necessity for additional meals, ability to work, dissatisfaction with working, and dissatisfaction with daily life subscales; all *p* < 0.05) and a trend towards a better score in the reflux subscale. This study included a large number of patients, and the results supported the use of PG. However, the following limitations made interpretation of the study results difficult: unbalanced disease stage (more advanced tumors underwent TG and extended LN dissection) and surgical approach (more patients in TG underwent open approach) between groups, reconstruction methods after PG not standardized, and cross-sectional study design, which meant that the survey was sent at various times after surgery.

The KLASS-05 randomized controlled study (NCT02892643) [23] recently reported results at the International Gastric Cancer Congress 2022. At 2 years after surgery, hemoglobin level changes did not differ between the laparoscopic TG and PG groups (*P* = 0.349), but a lower proportion of patients required vitamin B_12_ supplementation in the PG group (14.7% vs. 58.0%, *p* < 0.001). Moreover, late complications occurred more frequently among the TG group than the PG group (14.3% vs 10.8%, *p* = 0.738), and there was no increased incidence of reflux esophagitis after PG. The PG group had better physical functioning (*P* = 0.025) and social functioning (*P* = 0.031) at 2 years after surgery, suggesting an overall better QoL after PG, although the difference was not robust. QoL measurements were secondary outcomes in the KLASS-05 study and were only collected at the 2-year postoperative time point; therefore, the short-term QoL benefits of laparoscopic PG remain unknown.

The main focus of our proposed study will be the short-term QoL benefits of MIPG versus MITG, with the primary outcome being appetite level score at 3 months. We will utilize MDASI-GI [19], which is concise and written in easy-to-understand language, asking patients to score severity of symptoms experienced in the past 24 h, although it may not adequately identify less frequent symptoms that would be affecting patients’ quality of life. Although we added three fit-for-purpose question items to MDASI-GI for a total of 27 question items (MDASI-GI +), with its relatively small number of question items as compared to other validated questionnaires (e.g. EORTC QLQ-C30 plus STO22, with a total of 52 question items), MDASI-GI + may not detect details of postgastrectomy symptoms. However, after careful consideration of a choice of questionnaire to be used in this study, MDASI-GI + with its simple structure, which is expected to take less than 3 min for patients to complete, was considered an ideal questionnaire to be used in multiple languages and can be conveniently repeated to enable our longitudinal survey study.

This proposal is novel because it is the first international collaborative study as well as the first American study to prospectively investigate the benefits of MIPG with anti-reflux techniques. The research is culturally innovative because we will focus on PRO as the primary outcome, thus promoting a growing appreciation of the importance of patient-oriented care in the field of surgical oncology [24]. Theoretical benefits of PG, such as an improved hemoglobin level and reduced weight loss, become clinically significant only when QoL is improved, which can only be measured using PROs. The primary endpoint (appetite level) will be serologically validated with ghrelin level, which is an appetite-stimulating hormone that is known to decrease after TG [5]. Expected confounding factors that would affect postgastrectomy PROs, such as use of neoadjuvant therapy and different types of reconstruction after PG, will be carefully analyzed in sensitivity analyses. In addition, our study will determine the safety and feasibility of MIPG (including using a robotic approach) in the international setting. This transpacific collaboration between high-volume academic cancer centers will establish the foundation for future larger trials in the field of gastric cancer surgery.

## Data Availability

Not applicable.

## Abbreviations

P/GEJ: Proximal gastric and gastroesophageal junction
TG: Total gastrectomy
PG: Proximal gastrectomy
QoL: Quality of life
LN: Lymph node
MIPG: Minimally invasive PG
MITG: Minimally invasive TG
PRO: Patient-reported outcome
MDASI-GI: MD Anderson Symptom Inventory Gastrointestinal Cancer Module
